# Effect of Inhaled Cannabis for Pain in Adults With Sickle Cell Disease: A Randomized Clinical Trial

**DOI:** 10.1001/jamanetworkopen.2020.10874

**Published:** 2020-07-17

**Authors:** Donald I. Abrams, Paul Couey, Niharika Dixit, Varun Sagi, Ward Hagar, Elliott Vichinsky, Mary Ellen Kelly, John E. Connett, Kalpna Gupta

**Affiliations:** 1 Division of Hematology-Oncology, Department of Medicine, Zuckerberg San Francisco General Hospital, University of California, San Francisco; 2 Vascular Biology Center, Division of Hematology-Oncology-Transplantation, Department of Medicine, University of Minnesota Medical School, Minneapolis; 3 UCSF Benioff Children’s Hospital Oakland, Oakland, California; 4 School of Public Health, University of Minnesota, Minneapolis; 5 Hemtology/Oncology Division, Department of Medicine, University of California, Irvine

## Abstract

**Importance:**

Sickle cell disease (SCD) is characterized by chronic pain and episodic acute pain caused by vasoocclusive crises, often requiring high doses of opioids for prolonged periods. In humanized mouse models of SCD, a synthetic cannabinoid has been found to attenuate both chronic and acute hyperalgesia. The effect of cannabis on chronic pain in adults with SCD is unknown.

**Objective:**

To determine whether inhaled cannabis is more effective than inhaled placebo in relieving chronic pain in adults with SCD.

**Design, Setting, and Participants:**

This pilot randomized clinical trial included participants with SCD with chronic pain admitted to a single inpatient clinical research center for 2 separate 5-day stays from August 2014 to April 2017. Participants inhaled either vaporized cannabis (4.4% Δ-9-tetrahydrocannabinol to 4.9% cannabidiol) 3 times daily or vaporized placebo cannabis. Pain and pain interference ratings using the Brief Pain Inventory were assessed throughout each 5-day period. Participants with SCD and chronic pain on stable analgesics were eligible to enroll. A total of 90 participants were assessed for eligibility; 56 participants were deemed ineligible, and 34 participants were enrolled. Of these, 7 participants dropped out before randomization. Of 27 randomized participants, 23 completed both treatment arms of the crossover study and were included in the final per protocol analysis. Data analysis was completed in June 2019, with the sensitivity analysis conducted in April 2020.

**Interventions:**

Inhalation of vaporized cannabis plant (4.4% Δ-9-tetrahydrocannbinol to 4.9% cannabidiol) or placebo cannabis plant using a vaporizer 3 times daily for 5 days.

**Main Outcomes and Measures:**

Daily pain assessed with visual analog scale and Brief Pain Inventory.

**Results:**

A total of 23 participants (mean [SD] age, 37.6 [11.4] years; 13 [56%] women) completed the trial. The mean (SD) difference in pain rating assessment between the cannabis and placebo groups was −5.3 (8.1) for day 1, −10.9 (7.0) for day 2, −16.5 (9.2) for day 3, −8.9 (6.7) for day 4, and −8.2 (8.1) for day 5; however, none of these differences were statistically significant. There was no statistically significant mean (SD) difference in pain interference ratings between cannabis and placebo between days 1 and 5 for interference in general activities (day 1: 0.27 [0.35]; day 5: −1.0 [0.5]), walking (day 1: 0.14 [0.73]; day 5: −0.87 [0.63]), sleep (day 1: 0.59 [0.74]; day 5: −1.3 [0.8]), or enjoyment (day 1: 0.23 [0.69]; day 5: −0.91 [0.48]), but there was a statistically significant mean (SD) difference in decrease in interference with mood (day 1: 0.96 [0.59]; day 5: −1.4 [0.6]; *P* = .02). No differences in treatment-related adverse effects were observed. Use of concomitant opioids was similar during both treatment periods.

**Conclusions and Relevance:**

This randomized clinical trial found that, compared with vaporized placebo, vaporized cannabis did not statistically significantly reduce pain and associated symptoms, except interference in mood, in patients with SCD with chronic pain.

**Trial Registration:**

ClinicalTrials.gov Identifier: NCT01771731

## Key Points

**Question:**

Is inhaled cannabis with an approximately 1:1 ratio of Δ-9-tetrahydrocannabinol to cannabidiol a safe and effective adjunct to opioids in adults with sickle cell disease–related pain?

**Findings:**

This randomized clinical trial including 23 participants found that inhaled cannabis was safe. Inhaled cannabis was more effective than inhaled placebo in interference in mood, but there was no statistically significant difference in pain rating between cannabis and placebo.

**Meaning:**

These findings suggest that cannabis should be investigated further in larger and longer clinical trials in adults with sickle cell disease with chronic pain as an adjunct or alternative to opioids.

## Teaser

This randomized clinical trial examines the safety and efficacy of cannabis for pain in adults with sickle cell disease.

## Introduction

Cannabis is currently available for medicinal use in 33 states and the District of Columbia.^1^ Pain is the principle reason individuals report for accessing cannabis from dispensaries nationwide.^2^ A report by the National Academies of Sciences, Engineering and Medicine^3^ cited pain as the therapeutic indication with the strongest evidence base in the published medical literature. However, little data from controlled human clinical trials are currently available to support the widespread use of medicinal cannabis in painful conditions, including sickle cell disease (SCD).

Sickle cell disease is characterized by chronic pain with intermittent acute painful vasoocclusive crises. Recently, several new drugs have demonstrated effectiveness in preventing or decreasing the frequency of vasoocclusive crisis pain.^4,5^ However, therapies for chronic pain in SCD remain underinvestigated, although 55% of individuals with SCD report having pain more than 50% of the time.^6^ Opioids remain the mainstay for treatment, despite downsides that include constipation, pruritus, respiratory depression, and risk of addiction. The increasing incidence of opioid-associated deaths has further escalated opioid hesitancy among clinicians, potentially compromising the treatment of pain in SCD.^7^

An incomplete understanding of the mechanisms underlying SCD pain contributes to the lack of effective treatments.^8^ Pain in SCD is complex, demonstrating neuropathic as well as inflammatory characteristics.^9^ Likely contributors include systemic inflammation, neurogenic inflammation, oxidative stress, hypoxia and reoxygenation, vascular dysfunction, and end-organ damage. Preclinical studies suggest that cannabinoids may ameliorate pain and address the underlying pathophysiologic changes in SCD.^10,11^ Intraperitoneal CP55,940, a synthetic nonselective high affinity agonist of cannabinoid receptors 1 and 2 significantly reduced chronic and hypoxia–reoxygenation-evoked pain in HbSS-BERK sickle mice.^10,11,12^ These mice closely recapitulate clinical and pathophysiological features of SCD.

Cannabinoids have analgesic and anti-inflammatory properties and may ameliorate mast cell activation, leukocyte trafficking and adhesion, neurogenic inflammation, oxidative stress, endothelial activation, and hyperalgesia via cannabinoid receptors 1 and 2.^11,13^ Several trials have suggested that cannabis may effectively treat neuropathic pain.^1,14,15^ In addition to the analgesic effects of Δ-9-tetrahydrocannabinol (THC), the main psychoactive cannabinoid in cannabis, there are anti-inflammatory and analgesic properties purportedly associated with cannabidiol (CBD).^16,17^ These findings provided the basis for conducting this human proof of principle study of the safety and effectiveness of vaporized cannabis in adults with SCD with chronic pain.

## Methods

The study was approved by the institutional review boards at the University of California, San Francisco, and the University of Minnesota, as well as the Research Advisory Panel of California, Drug Enforcement Administration, Food and Drug Administration, and National Institute on Drug Abuse. Written informed consent was obtained from all participants prior to initiating the study. The study was conducted in the University of California, San Francisco, Clinical and Translational Science Institute’s inpatient clinical research center at Zuckerberg San Francisco General Hospital. This study is reported following the Consolidated Standards of Reporting Trials (CONSORT) reporting guideline.

### Selection Criteria and Study Participants

Adults with hemoglobin SS and chronic SCD-related pain receiving opioid analgesic therapy were enrolled between August 2014 and April 2017 (Trial Protocol and Statistical Analysis Plan in Supplement 1). All participants were using a stable pain medication regimen for at least 2 weeks. All participants were required to have prior experience smoking cannabis so they would know how to inhale and what neuropsychological effects to expect. Current users were asked to discontinue any cannabis use for 1 week prior to study admission. Because the Food and Drug Administration considered inhaled CBD to be a Novel Molecular Entity, participants were required to have prior CBD exposure so they would not be exposed to further risk. A negative pregnancy test result was required for women, and men and women were asked to use adequate birth control during the study. Exclusion criteria included severe coronary artery disease, uncontrolled hypertension, cardiac ventricular conduction abnormalities, orthostatic mean blood pressure drop of greater than 24 mm Hg, severe chronic obstructive pulmonary disease, history of renal or hepatic failure, evidence of clinically significant hepatic or renal dysfunction based on judgment of physician, active substance abuse, neurological dysfunction or psychiatric disorder severe enough to interfere with assessment of pain, current use of smoked tobacco products or a confirmed cotinine level, pregnant or breast-feeding women, or not practicing adequate birth control.

### Study Medication

The National Institute on Drug Abuse provided cannabis plant material containing 4.4% THC and 4.9% CBD as well as placebo cannabis from which the cannabinoids had been extracted. The study medications were vaporized in a vaporizer (Model #0100; Volcano) heated to 190 °C.^18^ This device inflates a bag with vapors derived from heating the cannabis plant material; this process should not be confused with inhalation of a heated oil from an e-cigarette, known as *vaping*. We previously demonstrated that this vaporization procedure results in plasma THC levels similar to that of smoked cannabis without significant exposure to carbon monoxide and other combustion products.^18^ To standardize doses, participants followed a uniform Foltin puff procedure.^19^ Participants self-titrated their doses but were encouraged to inhale at least 1 full bag of vapor.

### Study Timeline and Procedures

Eligible participants were admitted for 2 inpatient stays of 5 days and 4 nights in the clinical research center that were separated by at least 30 days. During 1 stay, participants inhaled vaporized cannabis 3 times daily, at 8 am, 2 pm, and 8 pm; during the other stay, they inhaled vaporized placebo cannabis on the same schedule.

Participants continued their outpatient analgesic regimen with additional inpatient analgesics prescribed as needed for increased pain. Participants who developed an acute painful crisis were transferred to the Zuckerberg San Francisco General Hospital emergency department for evaluation and admission as needed.

### Effects Monitoring

Participants scored their chronic pain on a visual analogue scale from 0 to 100 administered on arrival and repeated daily, 2 hours after morning drug inhalation, during the admission. The Brief Pain Inventory was administered on day 1 and repeated on day 5.^20^ Participants were evaluated for side effects by nursing staff every 4 hours while awake.

### Statistical Analysis

The study followed a crossover design. Each participant was exposed to a period of 5 inpatient days of either vaporized cannabis or vaporized placebo, then a month-long washout period out of the hospital, followed by a second 5-day admission with the opposite treatment. Thus, each participant was randomly assigned to the sequence active-placebo (indicating active drug in the first period and placebo in the second period), or placebo-active (indicating placebo in the first period and active drug in the second period). This design enabled each patient to effectively act as their own control, cancelling the effects of within-person variables such as sex, age, severity of sickle-cell disease, and other variables that may be related to the response to cannabis exposure.

#### Sample Size

Because this was a relatively small pilot study with many possible outcome variables of interest, it was designed to detect a one-half SD difference between active drug and placebo for any one of the variables of interest with 80% power. The target sample size was 35.

#### Randomization and Masking

Randomization order of cannabis and placebo was computer-generated by the study statistician and managed by an independent research pharmacist. Treatment was double-blind.

#### Within-Group Data Analysis

In a crossover design, the within-group analysis for a given outcome variable X is based on a 1-sample *t* test with the variable *X_A_* – *X_P_* computed for each person, where *X_A_* is the individual's response to the active drug *A*, and *X_P_* is that person’s response to the placebo *P*.

#### Between-Group Data Analysis

The between group comparisons (eg, sex, hydroxyurea use) were performed by taking the mean of the crossover difference, *X_A_* – *X_P_*, for a given outcome variable in a specific group over all 5 days for all participants within that group (eg, mean crossover pain difference for all women over all 5 days) and then performing a 2-sample *t* test to compare the means between different groups (eg, men vs women). While a crossover design cancels out within-person effects, thus reducing a major source of variability, there are some disadvantages, including that the usual practice, if there are carryover effects, is to analyze first-period data only, which loses the key advantage of the crossover structure and that the crossover analysis does not use the data if the person is missing the response to either drug *A* or drug *P*; that is, the crossover analysis for a given variable is restricted to people who have data from both periods. Further, in most crossover trials (including this one), it is necessary to impose a washout period between the 2 drug-exposure periods to eliminate the possibility of carryover effects. In this trial, the 2 drug-exposure periods were 5 days long, separated by a 1-month washout period with no exposure to the drug or placebo, which was deemed adequate to avoid carryover.

Analyses were performed using SAS statistical software version 9.4 (SAS Institute). *P* values were 2-sided, and statistical significance was set at *P* ≤ .05. Data analysis was completed in June 2019, with the sensitivity analysis conducted in April 2020.

## Results

### Study Participants

**Figure 1.**
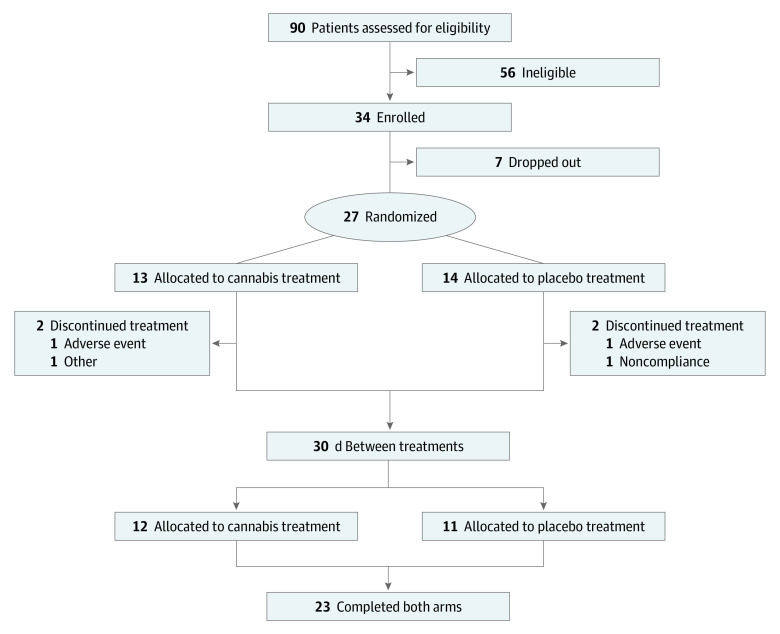
Consort Diagram

A total of 90 participants were assessed for eligibility. Among these, 34 participants met eligibility criteria and were enrolled (Figure 1). The most common reasons participants were screened out were SCD other than hemoglobin SS, inability to comply with the study visit calendar (eg, because of work or school schedules, childcare issues, or location too far removed from the study center), and failure to report for the screening visit after completing the telephone screening. Of 34 participants enrolled, 7 participants never started treatment: 6 participants developed scheduling conflicts that could not be resolved, and 1 participant was lost to follow-up between the date of enrollment and the anticipated first day of treatment. An additional 4 participants discontinued treatment prior to completion, including 2 participants because they had pain crises midintervention, 1 participant because of protocol nonadherence, and 1 participant because a nonstudy physician advised against further participation. Thus, 27 participants were treated or partially treated, and 23 completed both arms of the trial. The screened participants were predominantly African American, with 2 participants identifying as mixed race. Of 23 participants who completed both 5-day admissions, the mean (SD) age was 37.6 (11.4) years and 13 (56%) were women; 6 men and 9 women were receiving treatment with hydroxyurea (Table 1).

**Table 1.** Patient Demographics

Characteristic	Patients, No. (%)		
	Active-placebo (n = 11)	Placebo-active (n = 12)	Overall (n = 23)
Sex			
Men	6 (54.5)	5 (41.7)	11 (47.8)
Women	5 (45.5)	7 (58.3)	12 (52.2)
Age, mean (SD), y	41.7 (12.4)	33.8 (9.3)	37.6 (11.4)
Hydroxyurea use	7 (46.7)	8 (53.3)	15 (65.2)
Race/ethnicity			
Black	11 (100)	10 (83.3)	21 (91.3)
Other	0	2 (16.7)	2 (8.7)

### Pain

**Figure 2.**
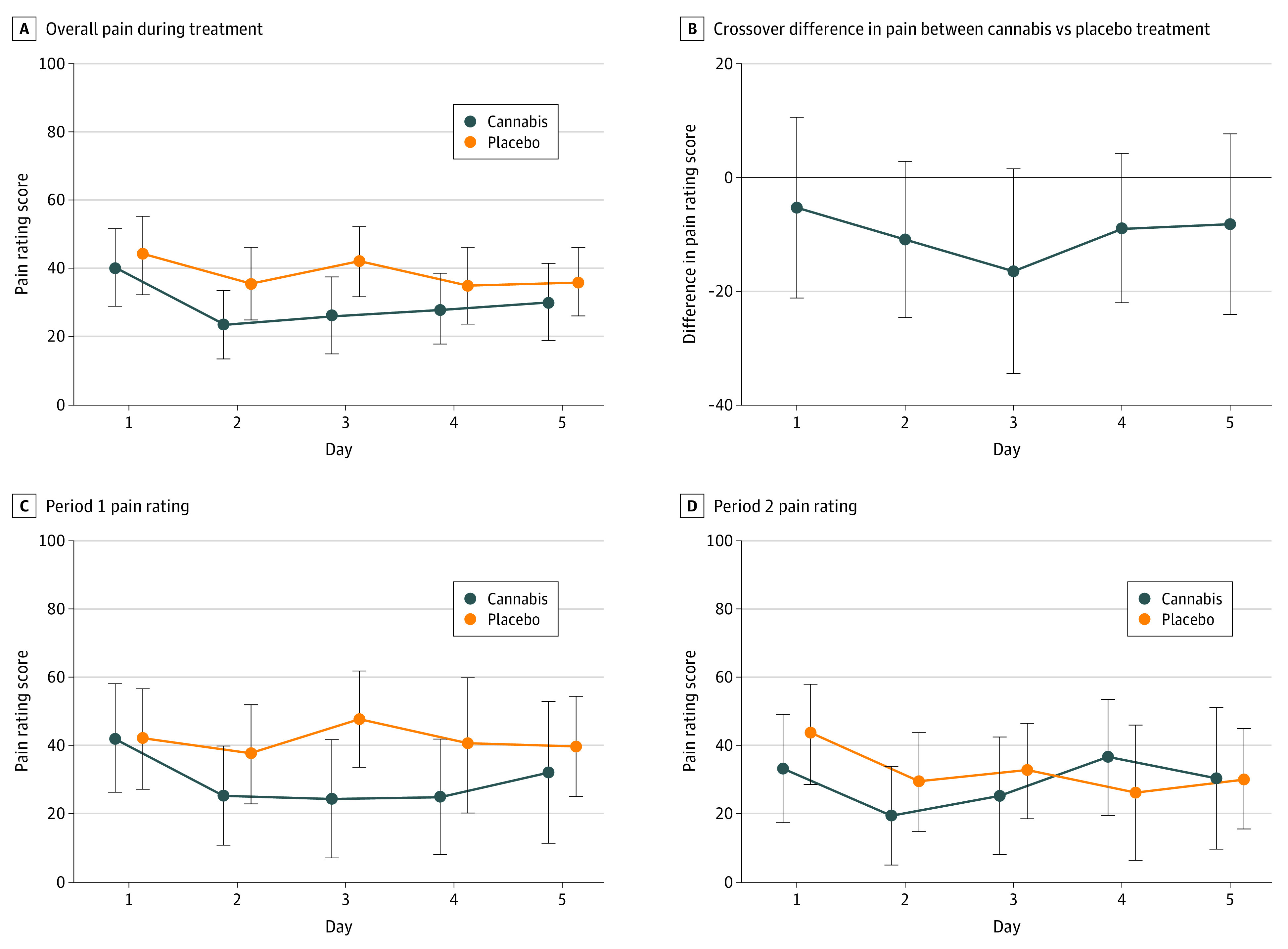
Daily Pain Rating During Cannabis and Placebo Treatment Participants completed a self-reported assessment of pain on the Drug Effects Questionnaire during each day of cannabis and placebo treatment. Pain was rated on a visual analog scale from 0 to 100. Dots indicate mean and whiskers, 95% CI.

**Figure 3.**
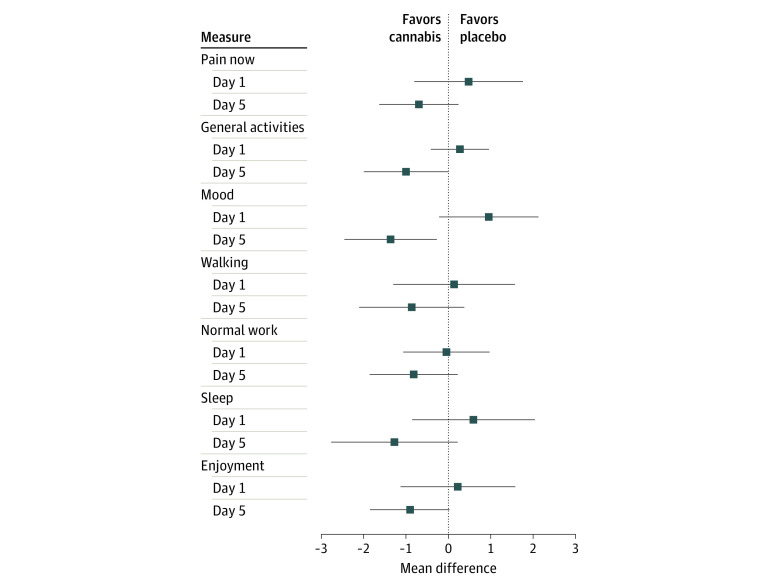
Difference in Pain Interference Ratings Between Cannabis and Placebo Treatment The brief pain inventory, a self-reported assessment of various chronic pain interference measures, was completed by each patient on days 1 and 5 of the cannabis and placebo treatment periods. Each of the interference criteria was rated on a scale from 1 to 10. Dots indicate mean and whiskers, 95% CI.

The mean (SD) difference in pain rating assessment using the visual analog scale data between the active and placebo groups was −5.3 (8.1) on day 1 (*P* = .51), −10.9 (7.0) on day 2 (*P* = .12), −16.5 (9.2) on day 3 (*P* = .07), −8.9 (6.7) on day 4 (*P* = .19), and −8.2 (8.1) on day 5 (*P* = .32) (Figure 2A and B). No statistically significant period effect was observed, as response to cannabis or placebo was similar irrespective of the order of intervention (Figure 2C and D). There were no statistically significant mean (SD) differences in pain interference ratings between cannabis and placebo between days 1 and 5 for interference in general activities (day 1: 0.27 [0.35]; day 5: −1.0 [0.5]), walking (day 1: 0.14 [0.73]; day 5: −0.87 [0.63]), sleep (day 1: 0.59 [0.74]; day 5: −1.3 [0.8]), and enjoyment (day 1: 0.23 [0.69]; day 5: −0.91 [0.48]). There was a statistically significant decrease in interference with mood (day 1: 0.96 [0.59]; day 5: −1.4 [0.6]; *P* = .02) (Figure 3). The mean differences in pain between cannabis and placebo treatment over all 5 days were compared based on sex and hydroxyurea use (eFigure in Supplement 2). There were no statistically significant mean (SD) differences for women vs men (−13.1 [12.9] vs −4.8 [10.2]; *P* = .63) or for hydroxyurea users vs nonusers (−4.1 [10.6] vs −18.45 [11.8]; *P* = .39).

### Opioid Use

Participants were generally using at least 1 opioid analgesic at study entry, which was maintained during their admissions, receiving breakthrough analgesics as needed. Hydromorphone was used by 10 participants, followed by oxycodone by 9 participants, hydrocodone by 7 participants, morphine sulfate by 6 participants, fentanyl by 2 participants, methadone by 2 participants, and oxymorphone by 1 participant. Two participants did not use any opioids, 9 participants used 1 opioid, 10 participants used 2 opioids, and 2 participants used 3 opioids. Among participants who used opioids, 13 participants required more morphine equivalents during the placebo admission compared with the cannabis admission, 3 participants required the same amount, and 5 participants required more opioids during the cannabis admission. The log morphine milligram equivalents were calculated for each study period because of the wide dosage range. The mean (SD) difference between the cannabis and placebo periods in this value was not significant (2.05 [0.21] vs 2.09 [0.22]; *P* = .20).

### Adverse Effects

Treatment was well tolerated. The most common side effect observed was sedation (Table 2). Adverse effects were generally mild and self-limited and scored less than 1 on a scale of 1 to 3. There were no significant differences between the mean aggregate (summed over all 5 days for each patient) adverse effect scores between the cannabis and placebo periods. One participant randomized to cannabis in period 1 developed an acute pain crisis and progression of liver failure that was deemed unrelated to study medication.

**Table 2.** Mean Aggregate Adverse Effects Scores During Cannabis and Placebo Treatment Periods

Adverse effect	Aggregate adverse effects score, mean (SE)	
	Cannabis	Placebo
Anxiety	0.61 (0.20)	0.96 (0.34)
Sedation	3.87 (1.03)	1.78 (0.63)
Disorientation	0.22 (0.18)	0.17 (0.10)
Paranoia	0.04 (0.04)	0.09 (0.09)
Confusion	0.09 (0.06)	0.17 (0.12)
Dizziness	0.43 (0.20)	0.26 (0.18)
Nausea	0.43 (0.31)	0.65 (0.36)

## Discussion

Cannabis use is relatively higher in people with SCD compared with the general population. Of 33 states that allow medical cannabis use, only 4 have included SCD as a qualifying condition. People with SCD continue to use cannabis, often from unapproved sources, thus increasing the risk of exposure to adverse effects. The US Centers for Disease Control and Prevention has increased public awareness of the risk of severe pulmonary disease associated with using electronic cigarette devices to vape tobacco and cannabis.^21^ In addition, recent multistate outbreaks of coagulopathy from synthetic cannabinoids have been traced to the presence of long-acting anticoagulant rodenticides in so-called *fake weed*.^22^ It is critical to evaluate the effectiveness of botanical cannabis in a prospective clinical trial, so that, if shown to be safe and effective in treating SCD pain, it can be made more widely available to eligible individuals, decreasing the risk of adverse consequences due to adulterated cannabis from unreliable sources.

To our knowledge, this is the first randomized, placebo-controlled clinical trial of vaporized cannabis in participants with SCD and chronic pain. To date, only 4 studies on cannabis use in individuals with SCD have been published, but all are surveys and/or retrospective analyses. In one, Knight-Madden et al^23^ aimed to determine the prevalence of cannabis smoking in the Jamaica Sickle Cell Cohort Study by surveying participants about their use in 2000 and again in 2004. By 2004, cannabis smoking was endorsed by 69% of men with SC and 62% of men with SS, with usage increasing 4-fold to 29% of women with SC and 19% of women with SS. Pain crises were counted, and Knight-Madden et al^23^ found no suggestion of different pain patterns between cannabis smokers and nonsmokers.

In the second study, a questionnaire was offered to adults with SCD at the Central Middlesex Hospital in London, prompted by investigators receiving anecdotal evidence of cannabis being used for analgesia from confidential accounts.^24^ During a 6-month period, 86 questionnaires were completed, and 31 people (36%) reported using cannabis, including 16 people who used cannabis for medicinal reasons, mainly to ameliorate acute and chronic pain and reduce analgesics. There was no evidence of more severe SCD in these individuals. Howard et al^24^ suggested that cannabis may relieve acute and chronic pain and decrease opioid analgesic use.

In a 2005 retrospective study^25^ in adult participants with SCD in Philadelphia, Pennsylvania, random urine testing was conducted over a period of 15 years. A total of 270 urine drug screen tests were performed among 72 participants; cannabinoids were found in 144 urine tests from 37 participants, including 26 men and 11 women. Participants with positive results stated that they used cannabis for pain relief, relaxation, and management of anxiety or depression, and these participants were significantly more likely to also have urine test results positive for benzodiazepine, cocaine, or phencyclidine. There was no difference in opioid use between participants who did or did not use cannabis. Increased admissions for vasoocclusive crises were reported in the cannabis cohort.^24^

A 2018 survey study^26^ of cannabis use in SCD involving 58 adults receiving care at a Yale medical facility in New Haven, Connecticut, found that 42% reported cannabis use within the past 2 years, with use more common in men (66%) than women (33%). Most patients (92%) said that they used cannabis for pain, and 79% of patients reported that cannabis use allowed them to use less prescription pain medicines.

In all of these studies, individuals obtained their own cannabis from unidentified sources and were surveyed retrospectively. To our knowledge, this study is the first prospective trial in which participants used vaporized cannabis from a validated source with a defined 1 to 1 ratio of THC to CBD under controlled conditions. In our prior pharmacokinetic interaction study,^27^ vaporized 3.5% THC cannabis was added to sustained release morphine or sustained release oxycodone in participants with chronic pain, and an estimated 25% reduction in pain was observed, although that study was not powered for pain as an end point and was not placebo controlled. In this study, most participants were using opioids, and the magnitude of enhanced analgesia with the addition of vaporized cannabis with a THC to CBD ratio of approximately 1 to 1 was 22%, which is comparable. Karniol et al^28^ reported in 1974 that CBD modulates THC effects, particularly the psychoactivity. In a randomized clinical trial of vaporized THC and CBD alone and in combination in frequent and infrequent cannabis users, Solowij et al^29^ reported that low doses of CBD enhanced the intoxicating effects of THC, while high doses of CBD reduced them. This suggests that CBD may similarly modulate some of the analgesic effects of THC.

### Limitations

This study has some limitations, including the small sample size and the short duration of treatment. Additionally, the imposed 3 times daily dosing schedule may not be the most ideal analgesic regimen. Ad libitum dosing may more closely reflect real-world usage and result in better relief of pain but usually is not incorporated into controlled clinical trials. We consider the vaporization device used to be a benefit, as it avoids the inhalation of combusted plant products, such as those produced from smoking a rolled-paper cigarette, and the dangers of inhaling oils inherent in the current vaping trend involving electronic cigarettes.

In the current climate of increased awareness of the ongoing opioid epidemic, it would have been encouraging if this study had demonstrated decreased use of chronic analgesics during the active cannabis vaporization phase. Epidemiological data has suggested that in states where medicinal cannabis is available, fewer analgesic prescriptions are written and a decrease in opioid-associated deaths has been observed.^30,31^ Our study’s small sample size and short duration may have contributed to the inability to demonstrate decreased opioid use among participants receiving the active drug compared with the placebo.

## Conclusions

This randomized clinical trial found that the cannabis used in this study was well tolerated. Adverse events were mild and equivalent to those reported by the placebo recipients. In contrast to many of the pharmaceuticals in the clinician’s daily armamentarium, cannabis has been consistently shown to be a generally safe intervention. People with SCD are often using multiple medications. Since no significant adverse effects were observed, this proof of principle study has the potential to encourage and guide future larger and longer investigations into the potential use of cannabis-based interventions in chronic pain that could help to attenuate the ongoing public health crisis related to opioid use.

## Notes

Supplement 1. **Trial Protocol and Statistical Analysis Plan**

Supplement 2. **eFigure.** Forest Plot

Supplement 3. **Data Sharing Statement**
