# Supplementary material for: Effect of Inhaled Cannabis for Pain in Adults With Sickle Cell Disease: A Randomized Clinical Trial
Source: JAMA Netw Open. 2020 Jul 17;3(7):e2010874. doi: 10.1001/jamanetworkopen.2020.10874 (PMC7368173; doi:10.1001/jamanetworkopen.2020.10874)
Supplement: Supplement 1. — Trial Protocol and Statistical Analysis Plan [file jamanetwopen-3-e2010874-s001.pdf]

Update 10/9/2014

SPECIFIC AIMS

Our primary objective is to assess whether inhaling vaporized cannabis ameliorates chronic pain in patients with sickle cell disease (SCD). As these patients will all be on chronic opioid analgesics, we will also assess the possible synergistic affect between inhaled cannabis and opioids. We will also assess the clinical safety of the concomitant use of cannabinoids and these opioids in patients with SCD by monitoring the short-term side effects associated with combined therapy.

Chronic pain conditions remain problematic, especially in adult patients with SCD (1). Although opioids are effective analgesics, dose-limiting side effects in the form of sedation, nausea and vomiting, and fear of dependence often limit their use at higher – and possibly more effective – doses. Of particular interest, however, is the potential for greater than additive analgesic effect of cannabinoids and opioids in combination that would allow for opioid analgesic effect to be achieved at lower dosages than are necessary alone (2–5), which could overcome problems with both tolerance and side effects for both drug classes. Safety data on the combination in humans is limited at this time, especially in patients with SCD. Among the plant's bioactive cannabinoids, delta-9-tetrahydrocannabinol (THC) is most known for its psychoactive effects, although analgesic effects have also been ascertained. Cannabidiol, a non-psychoactive cannabinoid, is becoming increasingly recognized as a potent anti-inflammatory and analgesic that may have a unique place in the armamentarium of potential pain medications. As patients with SCD may turn to cannabis to augment the effects of their opioid analgesics and for possible anti-inflammatory effects to alter disease progression, data on the clinical safety and possible effectiveness of the combinations should be evaluated in a controlled proof of principle setting. Hence, we propose the following specific aims:

Aim 1: To determine the effects of inhaling vaporized cannabis on chronic pain in patients with SCD.

Hypothesis 1: Cannabis will significantly reduce chronic pain in patients with SCD.

We will test this hypothesis by conducting a series of 5-day, inpatient evaluations in 35 SCD patients with chronic pain. We will obtain a 5-day pain diary prior to admission to the Clinical Research Center (CRC) to establish a baseline of pain. Participants will then be assigned to inhale either vaporized cannabis of mixed THC/CBD content (4.7% THC/5.1% CBD) or placebo cannabis (0% THC/0% CBD). Participants and personnel will be blinded as to assignment. Pain will be evaluated during the 5-day inpatient exposure. Participants will be asked to participate in two such 5-day sessions separated by at least a month washout so that each will be exposed to the two experimental conditions.

Aim 2: To determine the short-term side effects associated with the co-administration of opioids and inhaled cannabis for SCD pain. Use of opioids is associated with dose-limiting side effects, including sedation, constipation, nausea and vomiting, and fear of dependence. Use of smoked cannabis has also been associated with a variety of side effects, including sedation, paranoia and dysphoria. It is possible that cannabis may potentiate similar side effects associated with the use of both drugs. It is also possible that the combination of opioids and inhaled vaporized cannabis may be associated with a unique and different side effect profile in patients with SCD. Cannabinoid receptor agonists act on pathways that partially overlap with those activated by opioids, but through pharmacologically distinct mechanisms. This study will help elucidate the short-term safety issues associated with the use of cannabis among patients prescribed opioids for SCD pain.

Hypothesis 2: Cannabis will significantly alter the short-term side effects experienced by patients who take opioids for SCD.

This hypothesis will be tested by administering a battery of patient-reported outcome questionnaires measuring side effects and mood to the participants during the two CRC admissions described above.

Aim 3: To determine the short-term effects of inhaled cannabinoids on markers of inflammation and disease progression in patients with SCD. Mouse models of SCD suggest that cannabinoid receptor agonists may have beneficial effects on markers of inflammation and disease progression. To date, this has not been investigated in a human SCD population.

Hypothesis 3: Inhaled cannabis will significantly alter markers of inflammation and disease progression in patients with SCD compared to placebo.

This hypothesis will be tested by collecting blood and urine samples for markers of inflammation and disease progression at the time of CRC admission on day 1 and again on day 5 after exposure to inhaled cannabinoids. The same samples will be collected one week after each inpatient stay. Blood and urine will be frozen and sent to collaborators at the University of Minnesota for batch analysis at the conclusion of the project.

A. BACKGROUND AND SIGNIFICANCE

A 2011 Institute of Medicine report- **Relieving Pain in America**- estimates that chronic pain affects nearly 116 million American adults, a staggering number that surpasses those affected by heart disease, cancer and diabetes combined (6). In addition, the report concludes that chronic pain costs between $560 billion and $635 billion annually in both medical expenses and lost productivity. Although there have been some recent therapeutic advances, many patients with chronic pain become resistant to conventional medical treatments or suffer adverse effects from widely-used prescription medications, such as non-steroidal anti-inflammatory agents or opiates, that have high addictive potential.

B.1 Chronic pain in sickle cell disease: Sickle cell disease is the most common genetic disorder in the United States, affecting more than 80,000 people, the majority of whom are African American. Most of the research on pain is SCD has focused on children with acute pain associated with sickle cell crisis. Little is known about the occurrence and characteristics of chronic pain, especially in adults with SCD. One literature review suggested that chronic pain occurs in at least 29% of adults with SCD, most frequently in adults 25 to 44 years of age (7). Chronic pain in adults with SCD can occur from disease complications such as avascular necrosis, ankle ulcers or acute pain superimposed on chronic pain. Many SCD patients with chronic pain become opiate-dependent. As individuals who experience SCD are often under-served, their pain is frequently under-treated resulting in frequent emergency room visits, hospitalizations, increased medical costs and lost work productivity. Effective mechanisms of ameliorating chronic pain in this population are needed. If these interventions also serve to decrease inflammation and impact markers of SCD progression they would be of even greater value.

B.2 Biological interaction between cannabinoids and opioids: Synergism between opioids and cannabinoids has been postulated and subsequently demonstrated in a number of animal models. The antinociceptive effects of morphine are predominantly mediated by mu receptors but may be enhanced by delta-9-tetrahydrocannabinol activation of kappa and delta opioid receptors (8). It has been further postulated that the cannabinoid:opioid interaction may occur at the level of their signal transduction mechanisms (9,10). Receptors for both classes of drugs are coupled to similar intracellular signaling mechanisms that lead to a decrease in cAMP production by way of G protein activation (10–12). There has also been some evidence that cannabinoids might increase the synthesis or release of endogenous opioids, or both (3,4,11,13).

B.2.1 Biological interaction between cannabinoids and morphine in animals: Welch and her colleagues have been at the forefront of describing the pharmacologic antinociceptive synergy of delta-9-tetrahydrocannabinol and morphine in numerous experiments. They initially demonstrated that intracerebroventricular or intrathecal administration of inactive doses of THC greatly enhanced the antinociceptive effect of morphine using the mouse tail-flick model (9). They subsequently demonstrated similar enhancement using oral and subcutaneous doses of THC, concluding that morphine's potency was significantly increased, regardless of the routes of THC and morphine delivery (3). It has been suggested that

to explain the greater than additive antinociceptive effect of the combination, a point of interaction must be shared by both drugs. It was hypothesized that this may occur via an intracellular second-messenger system (5). Again, cAMP and calcium modulation were examined, but the results appeared inconclusive (12). Cichewicz recently concluded that the synergy observed with THC and morphine most likely results from enhanced activation of the opioid receptor cascade (5).

B.2.2 Biological interaction between cannabinoids and oxycodone in animals: No animal studies have been conducted on the interactions between cannabinoids and oxycodone. However, relevant to the proposed clinical trial, Cichewicz et al. found increased potency of several other mu opioids, including hydromorphone and oxymorphone (a metabolite of oxycodone), when administered after oral delta-9-THC in the mouse tail-flick model (4). These investigators have been encouraged by their preclinical data to the point where they suggest that the administration of low-dose delta-9-THC in conjunction with low doses of opioids seems to be an alternative regimen for enhancing the pain-relieving effect of opioids, without the side effects characteristic of either drug (4,10).

B.3 Side effects of opioids and cannabinoids

B.3.1 Morphine: The most frequently observed side effects of morphine are constipation, light-headedness, dizziness, sedation, nausea, vomiting, sweating, dysphoria and euphoria. The most serious side effects associated with the use of morphine are respiratory depression and cardiovascular stimulation. Because of delay in maximum central nervous system effect, rapid administration may result in overdosing. While low doses of morphine have little effect on cardiovascular stability, high doses are excitatory (14).

In a narrative review, Warfield examined 10 well-controlled studies of CR morphine tablets in patients with cancer pain (15). Scores obtained in these studies used CAT 3-, 4-, 5-, or 7-point scales and were converted to corresponding values on a common 10-point scale. The highest average scores were for sedation, with a mean score of 2.5, which was transient over 48 to 72 hours. Other average scores were for constipation, with a score of 2.0; nausea, with a score of 0.9; dizziness, with a score of 0.7; emesis, with a score of 0.3; dryness of the mouth, with a score of 2.4; followed by agitation, with a score of 1.8. Of the 16 patients treated with CR morphine in Study 1, in which 18 patients were randomized and 16 patients completed study, 44% had nausea and emesis, and 44% reported dizziness on 1 or more occasions. In Study 8, in which 44 patients were randomized and 36 patients completed study, overall sedation occurred in 16.7% of patients, dry mouth in 14.3%, constipation in 12.5%, nausea in 11.8%, urinary retention in 7.7%, and emesis in 2.6%. There were no cases of respiratory depression. In study 10, 24 patients were randomized and 10 completed the study. Side effects reported by this group were headache, bone pain, swollen extremities, twitching, and excitement. It was also noted that patients with colon carcinoma with abdominal carcinomatosis developed constipation that lasted for 3-5 days.

B.3.2 Oxycodone: Side effects of oxycodone are similar to those of morphine. The most common side effects are drowsiness, lightheadedness, nausea, vomiting, pruritis, constipation, and sweating accompanied by hot flashes (16,17). Oxycodone use is associated with fewer opioid-induced hallucinations, less nausea and vomiting, and more constipation compared to morphine (16,18–20). In a study by Maddocks et al., patients with morphine-induced delirium experienced significantly improved mental status after substituting oxycodone for morphine. Patients in this study also experienced less nausea and vomiting on oxycodone compared to morphine (21,22).

B.3.3 Cannabinoids: Acute affects attributed to smoking cannabis include psychological, psychomotor, cognitive and cardiovascular effects. Acute psychological effects include anxiety, dysphoria, panic and paranoia. These have been observed primarily in naïve users, but are occasionally reported by experienced users who receive a much larger than intended dose (23). Psychomotor impairment has been attributed to smoking cannabis based on the results of several case-control studies that have observed an increase in motor vehicle accidents among those who smoke cannabis (24); however, findings from experimental studies of psychomotor impairments have been mixed. For example, reaction time has been reported to be slower following acute cannabis exposure in some experiments (25-27); but in other experiments, reaction time has

been shown to be unaffected (25,28). Observed cognitive effects of smoked cannabis include impairment of short-term memory, decreased attention span, decreased verbal facility, and slower problem-solving. These affects are dose-related and occur only for the duration of intoxication (24,29). Similarly cardiovascular effects are observed during intoxication including an increase in heart rate and usually some increase in blood pressure (24,25). Inhaled cannabis vapors have a similar profile of effects. **These data are derived from studies of THC-containing cannabis; the risks of cannabidiol have not yet been fully documented. Though hardly conclusive, prior animal studies have suggested that clastogenicity and impaired spermatogenesis may result from CBD-rich cannabinoid use. We are thus requiring study participants to have prior experience with cannabidiol-rich cannabis.**

B.3.4 Side effects associated with co-administration of opioids and cannabinoids: Few studies have investigated the objective or subjective effects associated with the co-administration of opioids and cannabinoids in humans. In two studies of healthy volunteers, co-administration of opioids and cannabinoids appeared to increase the cardiovascular effects, but to decrease or not affect the subjective effects of each of these drugs taken individually. In a 1975 study of 15 healthy volunteers (30), co-administration of 1.0 mg/70 kg iv of oxymorphone and 134 mug/kg of oral delta-9 THC increased participants' heart rate and cardiac index and decreased participants' CO_2 ventilation and total peripheral resistance, but did not increase participants' sedation or anxiety, or induce hallucinations. In a more recent study of 12 cannabis-naïve healthy volunteers (31), oral administration of 30 mg of morphine and 20 mg of delta-9 THC reduced participants' systolic and diastolic blood pressure and oxygen saturation, but did not alter participants' heart rate. In addition, co-administration of morphine and THC increased participants' sleepiness, but decreased many of the psychotropic side effects of THC (e.g., euphoria, hallucinations, and confusion), as well as nausea and vomiting associated with the use of morphine. Our own studies showed no increase of adverse effects when inhaled vaporized cannabis was combined with stable opioid doses in patients with chronic pain (see section C).

B.4 Anti-inflammatory effects of cannabinoids: Through interaction predominantly with the CB2 receptor, cannabinoids may have effects on the immune system that leads to anti-inflammatory effects. The CB2 receptor was originally detected in macrophages and the marginal zone of the spleen, with the highest concentration reported in natural killer cells and B lymphocytes, suggesting a potential role in immune function. Part of the analgesic effect of cannabinoids is felt to be related to peripheral anti-inflammatory effects. Cannabidiol (CBD), a non-psychoactive cannabinoid, is felt to have potent anti-inflammatory and analgesic activities (32-34). CBD has low affinity for the CB1 and CB2 receptor for its effects; it may interact with the endocannabinoid system acting as an inhibitor of fatty acid amide hydrolase, the major enzyme responsible for endocannabinoid breakdown. CBD exerts multiple pharmacological actions in the central nervous system and the periphery including analgesic, anti-inflammatory, antioxidant, neuroprotective and pro-apoptotic. CBD may have utility in treatment of pain, neurodegenerative diseases, ischemia and cancer – hence potentially quite a potent therapy for patients with SCD.

B.5 Significance: Chronic pain in sickle cell disease is a major health problem in this population (1). Many patients continue to experience chronic pain and episodic acute crises despite opioid maintenance. An increasing number of states across the country have established provisions for patients to utilize cannabis for medicinal purposes. It is likely that significant numbers of patients with various medical conditions utilizing opioid analgesics might self-medicate with inhaled cannabis. No clinical information exists on the potential effectiveness of adjunctive cannabis in reducing chronic pain, decreasing opioid use, decreasing vaso-occlusive crises and decreasing utilization of medical care in patients with SCD.

This study will also provide important information on the potential for cannabinoids to impact markers of inflammation and disease progression in SCD. Being a more potent anti-inflammatory, the presence of CBD cannabis being evaluated will likely enhance these effects over strains of cannabis that contain THC alone. If the data supports this hypothesis, inhaled cannabis may not only be a useful adjunct in the treatment of SCD-related chronic pain, but it may also serve to decrease episodes of acute pain and ameliorate the manifestations of SCD overall.

C. PRELIMINARY STUDIES

C.1 Preliminary studies of cannabis in HIV patients

We completed a study of the short-term effects of cannabinoids in patients with HIV infection in 2000. This National Institute on Drug Abuse (NIDA)-supported study enrolled 67 patients with HIV infection on a protease inhibitor-containing antiretroviral regimen to investigate the potential for interaction between cannabis and the protease inhibitor or cannabis and the immune system that may lead to perturbation of HIV viral load (35).

Participants were randomized to one of the following three arms: (1) a 3.95% tetrahydrocannabinol cannabis cigarette three times daily before meals, (2) a 2.5 mg dronabinol capsule (delta-9-tetrahydrocannabinol) three times daily before meals or (3) a placebo capsule three times daily before meals. Participants were housed for 25 days in the General Clinical Research Center (GCRC) at SFGH. The primary endpoint was change in HIV RNA level at the end of the 21-day experimental period. Measurements included HIV RNA levels, T lymphocyte subsets, and pharmacokinetic analyses of the protease inhibitors. Of 67 study participants randomized, 62 were evaluable for the primary endpoint: 20 randomized to cannabis, 22 to dronabinol, and 20 to placebo. Baseline HIV RNA level was <50 copies/mL for 36 subjects (58%) and the median CD4+ lymphocyte count was 340 x 10^9 cells/L.

Pharmacokinetic investigations of the cannabinoids in this trial demonstrated that the participants who smoked cannabis achieved significantly increased 6-hour area under the curve and maximum concentration of THC compared to the oral delta-9-THC recipients (16). Trough levels of plasma delta-9-THC were obtained just prior to the second cannabis cigarette smoked on day 14. Additional levels were drawn 2 minutes, 60 minutes and 6 hours after smoking. For the oral dronabinol subjects' levels after the trough were obtained at 2, 4 and 6 hours after the dose. The cannabis arms achieved median C_{max} and AUC_6 values of 141 ng/ml and 77 ng/ml•hr compared to 1.1 ng/ml and 4.1 ng/ml•hr in the dronabinol recipients ($p<0.001$ for all comparisons between cannabis and dronabinol).

Overall, there was no change in the level of HIV RNA in any of the three groups over the study period. There was a suggestion of an increase in the number of CD8+ T lymphocytes in the group smoking cannabis (36). No clinically significant pharmacokinetic interactions were seen between the cannabinoids and the protease inhibitors (37). On the basis of these findings we felt that it was safe to continue to investigate the utility of smoked cannabis in patients with HIV-related painful peripheral neuropathy.

C.2 Cannabis vaporization

We conducted an investigation of the Volcano vaporizer as a smokeless cannabis delivery system (38). The vaporizer device heats the cannabis to a temperature of approximately 190 degrees F, below the level of combustion. A fan in the device inflates a balloon-like reservoir from which the vaporized cannabis can be inhaled. In this trial, healthy volunteers aged 25-45 were admitted for 6 days to the GCRC at SFGH. On each of the next six days, subjects either smoked or vaporized half of a NIDA 1.7%, 3.4% or 6.8% THC containing cigarette. Pharmacokinetic sampling was obtained to create concentration versus time curves. Participants were evaluated for physiologic and psychological effects of smoking versus vaporization of the cannabis. Eighteen participants (15 men and 3 women) completed the 6-day inpatient study. The peak plasma concentrations and six-hour area under the plasma concentration-time curve of THC after inhalation of vaporized cannabis were similar to those of smoked cannabis. Carbon monoxide levels were substantially reduced with vaporization. Neuropsychologic effects were equivalent and participants expressed a clear preference for vaporization as a delivery method. No adverse events were observed.

C.3 Preliminary studies of cannabis in pain

We have conducted a study of smoked cannabis in patients with HIV-related peripheral neuropathy supported by the University of California Center for Medicinal Cannabis Research with cannabis supplied by NIDA (39). Initially sixteen patients with HIV-related peripheral neuropathy were enrolled in the pilot phase of the trial. Subsequently we enrolled 50 patients in a randomized, placebo-controlled trial that also included the

heat-capsaicin experimental pain model. 50 patients completed the entire trial. Smoked cannabis reduced daily pain by 34% (median reduction; IQR=-71, -16) compared to 17% (IQR=-29, 8) with placebo ($p=0.03$). Greater than 30% reduction in pain was reported by 52% in the cannabis group and by 24% in the placebo group ($p=0.04$). The first cannabis cigarette reduced chronic pain by a median of 72% compared to 15% with placebo ($p<0.001$). Cannabis reduced experimentally-induced hyperalgesia to both brush and von Frey hair stimuli in the heat-capsaicin model ($p\leq0.05$). No serious adverse events were reported.

We also conducted a classic pharmacokinetic interaction study looking at the interaction between inhaled vaporized cannabis and opioids in patients with chronic pain supported by NIDA (40). Twenty-one subjects were enrolled (11 oxycodone, 10 morphine). Pharmacokinetic samples were drawn on day 1 on the chronic opioid dose prior to exposure to inhaled cannabis. Repeat sampling was obtained on day five after exposure to inhaled cannabis three times daily. Pharmacokinetic analysis revealed a non-significant decrease in the maximal plasma concentration and area under the curve for morphine, with no changes in oxycodone kinetics after exposure to vaporized cannabis. Time to maximal concentration tended to be during cannabis treatment. Pain was significantly lower with the addition of vaporized cannabis. The mean pain score decreased 11.2 (32%) in the morphine participants from day 1 to day 5 and 10.3 (24%) in the oxycodone participants ($p< 0.001$ for both). There were no serious adverse effects noted. Participants underwent continuous pulse oximetry monitoring throughout the night to rule out respiratory suppression if the cannabis had increased opioid plasma concentrations. One patient with SCD was enrolled in the pharmacokinetic trial. Her baseline pain on day 1 was rated as 32 on a 0-100 visual analogue scale; baseline pain on day 5 was reported as 23 (a 28% reduction). She experienced no adverse experiences.

D. RESEARCH DESIGN AND METHODS

D.1 Overview of study design: We propose to conduct a proof of principle investigation of the safety and potential effectiveness of inhaled vaporized cannabis when added to a stable analgesic regimen in SCD patients with chronic pain. The study will be conducted in the Clinical Research Center (CRC) at San Francisco General Hospital (SFGH). The inpatient setting permits us to rigorously assess the safety by way of closely observed nurse monitoring for potential side effects and the effectiveness pain intensive collection of pain data. It also permits us to collect and preserve specimens for future analysis of markers on inflammation and SCD disease progression.

The study will be comprised of two 5-day inpatient intervention periods in the CRC, with a brief outpatient follow-up visit one week after each inpatient stay. Participants will complete a 5-day daily pain diary prior to CRC admission to establish an outpatient baseline. Subjects will attend a screening visit where they will be screened for eligibility criteria. Research staff will then obtain informed consent and enroll interested and eligible subjects.

On Day 1 of each admission, subjects will provide blood for baseline markers of inflammation and SCD disease progression. Pain will be assessed using the Brief Pain Inventory as well as a visual analogue scale. At 2 pm on Day 1, participants will inhale 0.7 grams vaporized cannabis. The participant and CRC staff will be blinded as to the nature of the cannabis (mixed THC/CBD or placebo). On Days 2 through 4 subjects will inhale cannabis at 8am, 2pm and 8pm. On Day 5, subjects will inhale cannabis at 8am. Subjects will continue taking their pre-study analgesic medications (e.g., opioids, gabapentin, amitriptyline, NSAID) at a stable dose while in the study, which will be recorded daily. If a subject requires additional analgesia during the inpatient pilot study, supplemental therapy will be given and the dose recorded.

On Day 5, repeat specimens for markers of inflammation and sickle cell disease progression will be obtained. Participants will complete the Brief Pain Inventory again. The pain visual analogue scale will be completed daily throughout the study and finally on Day 5. One week after discharge, participants will return for a brief outpatient visit, during which a further set of specimens will be collected for biomarker testing.

D.2 Study population: The study will enroll adults on opioid analgesics for chronic pain due to their sickle cell disease. Eligible subjects will be experienced cannabis users (i.e. have tried at least 6 times in their life) in order to avoid exposing subjects to substances they might consider objectionable and to exclude subjects who might not inhale vaporized cannabis appropriately and thus receive an inadequate dose of study treatment. Although all subjects will have prior cannabis exposure, they will be given information about the range of subjective effects they may experience from cannabis, as well as techniques to assist subjects in coping if the side effects are in any way disturbing or disorienting. Subjects will be asked to abstain from using cannabis for 7 days prior to their scheduled CRC admissions.

D.2.1 Eligibility criteria

Inclusion criteria

1. Sickle cell disease
2. Ongoing opioid analgesic therapy for chronic sickle cell disease-associated pain.
3. Subjects must be on a stable dose of opioid analgesic medication for at least 2 weeks before enrollment.
4. Current other analgesic medications will be maintained during the study. The subject must have been on a stable medication regimen for at least 2 weeks.
5. All men and women in this study must agree to use adequate birth control during this study. Acceptable barrier birth control methods are a male condom, female condom, diaphragm, or intra-uterine (IUD).
6. All women of reproductive potential (who have not reached menopause or undergone hysterectomy, oophorectomy, or tubal ligation) must have a negative urine β-HCG pregnancy test performed before initiating the protocol-specified medication.
7. Prior history of use of cannabis. Subjects must have smoked cannabis on at least 6 occasions in their lifetime prior to enrollment**, and must have used cannabis containing cannabidiol in the past**.
8. Subjects will self-report abstaining from smoking or ingesting cannabis for one week prior to their enrollment into the study.
9. Able to understand and follow the instructions of the investigator, including completing the pain intensity rating scales.
10. Karnofsky Performance Scale $\geq$60.
11. Able and willing to provide informed consent.
12. Able and willing to spend 5-days and 4 nights in the Clinical Research Center at SFGH, two times during the study period.

Exclusion criteria

1. Severe coronary artery disease, uncontrolled hypertension, cardiac ventricular conduction abnormalities, orthostatic mean blood pressure drop greater than 24 mmHg, severe chronic obstructive pulmonary disease.
2. History of renal or hepatic failure.
3. Evidence of clinically significant hepatic or renal dysfunction based on judgment of physician.
4. Active substance abuse (e.g., alcohol or injection drugs).
5. Neurologic dysfunction or psychiatric disorder severe enough to interfere with assessment of pain or sensory systems.
6. Current use of smoked tobacco products.
7. Women who are pregnant or breast-feeding may not take part in this study.
8. Unable to read or speak English.

D.2.2 Subject recruitment: Subjects will be recruited from the Sickle Cell Clinic at SFGH, from Sickle Cell Clinics throughout northern California and from paid advertisements placed in the local print media.

D.3 Study procedures

D.3.1 Screening visit: The study coordinator will arrange a screening visit with potential subjects. At the screening visit, prospective subjects will review the consent form in detail with the study coordinator who will answer all questions before inviting the patient to sign the consent form. It will be stated that participation in research is voluntary and that subjects have the right to decline to participate or withdraw at any point in the study without jeopardy to their medical care. Subjects will also be asked to sign the UCSF Subject's Authorization for Research Access to Health Information and will be instructed that they may withdraw their authorization for this study to use their personal health information by contacting Dr. Abrams in writing to inform him of their decision. If subjects withdraw their authorization, the information already collected may continue to be used, to maintain the integrity of the study. If they choose not to sign this consent form, the investigator cannot use information from their medical records and they cannot participate in this research study. If a subject agrees to participate, the subject will sign the main consent form as well as the UCSF Subject's Authorization for Research Access to Health Information and a photocopy of the signed consent forms with the Experimental Subjects' Bill of Rights will be given to the subject. The protocols will receive approval from the Institutional Committee on Human Research of the University of California, San Francisco prior to implementation. If subjects are eligible and consent to continue, information on the subject's medical history will be collected and they will continue with the next phase of the study.

D.3.2 Inpatient phase (5 days)

1. Subjects will be admitted to the CRC at SFGH for a total of five days and four nights. During this time, they will not leave the hospital or be allowed to have visitors.
2. On the first day, subjects will have a brief physical. Female study subjects who are able to have children will have a urine pregnancy test performed. If the specimen is positive, she will be asked to leave the study.
3. On Day 1, blood will be collected, frozen and stored for future determination of markers of inflammation and SCD disease progression to be performed at the University of Minnesota collaborator's laboratories.
4. Subjects will have vital signs (heart rate, blood pressure and respiration rate) taken three times daily.
5. Subjects will complete the Brief Pain Inventory and Drug Effects Questionnaire prior to cannabis administration on Day 1. They will score their chronic pain on a 0-100 visual analogue scale and repeat that every two hours while awake.
6. Starting at noon of Day 1, subjects will inhale one cannabis cigarette vaporized three times daily in the Volcano vaporizer (8am 2pm 8pm). The subject and CRC staff will be blinded as to the nature of the cannabis (mixed THC/CBD or placebo). On Day 5, subjects will vaporize the final cigarette at approximately 8am. They will be instructed by the nursing staff in how to inhale the vaporized cannabis from the Volcano vaporizer in a standardized manner and will be given information about the different effects that they may experience when inhaling cannabis. Subjects will be instructed to use the uniform puff procedure, described by Foltin (41), in which the vapor is inhaled once a minute for 5 minutes as tolerated or until it is consumed. Standardization of inhaled dose and has been used successfully in all of our inhaled cannabis trials to date. An alternative would be to allow subjects to titrate intake to achieve a desirable effect; however, employing the Foltin procedure allows for some standardization of inhaled dose. CRC nursing staff will observe all subjects while they smoke and record the number of puffs. Subjects will be housed in a room with a fan ventilating to the outside.
7. On Day 5, subjects will inhale vaporized cannabis at 8am. Blood will be drawn for markers of inflammation and SCD disease progression. Patients will complete the Brief Pain Inventory and Drug Effects Questionnaire.
8. On all five days, subjects will be evaluated for side effects three times a day (8:30 am, 2:30 pm, 8:30 pm) using the Community Consortium Side Effects form.
9. After the blood drawing and data collection are completed on Day 5, subjects will be discharged. Subjects will be asked to either have someone pick them up or be given a taxi voucher to go home.
10. Subjects will be invited to return for a second similar admission at which time they will inhale the other cannabis preparation. The return admissions will be scheduled at least one month after the first. Participants will be again asked to refrain from using cannabis for 7 days prior to CRC admission.

D.3.2.1 Outpatient blood draw: Subjects will be asked to return to the CRC, one week after discharge from the inpatient stay, for a brief visit in which an additional set of blood and urine samples will be collected for markers of inflammation and SCD disease progression.

D.3.3 Reimbursement of subjects: Subjects will be reimbursed $20 for their screening visit, $50 per day for each of the hospitalization days, and $20 for the follow-up visits for specimen collection, for a total of $560. Subjects who do not complete the full course of the study will be reimbursed on a prorated fashion for the amount of time that they have participated. These reimbursement rates are consistent with other CRC studies conducted at SFGH. Subjects will be reimbursed by check, approximately one month after completing the study. They may choose to pick up the check or to have it mailed. A subject must provide their home address and social security number to receive payment for the study. The amount of payment may be reportable by the University of California, San Francisco, for income tax purpose.

D.3.4 Reporting of adverse events: For the purpose of study monitoring and analysis, all Adverse Events (AEs) at a toxicity Grade 3 or higher associated with use of the study drug will be considered Serious Adverse Events (SAEs). All Serious Adverse Events will be recorded on the Division of AIDS Regulatory Operations Center Serious Adverse Experience form and reported to the U.S. Food and Drug Administration (FDA). Serious adverse experiences are defined as a subset of those adverse events (including deaths) that are possibly related to the study treatment and require reporting to the FDA. Internal reporting procedures have been developed for timely and accurate reporting of serious experiences in order to monitor subject safety, to comply with FDA regulations, and to disseminate safety information to our institutional review board. If a subject develops a Grade **3 or** 4 serious adverse event they will be removed from the study treatment at that time. Subjects may withdraw from the trial at any time they wish.

D.4 Supply and storage of study drug

D.4.1 Supply: The cannabis required for this study will be provided by the National Institute of Drug Abuse (NIDA). Cannabis grown under contract with NIDA will be supplied to the Research Triangle Institute (Research Triangle Park, North Carolina) where the cannabis will be prepared. CRC nursing staff will weigh the cannabis cigarettes immediately before they are administered to subjects, and will retain and return all leftover material to the pharmacy for accounting. The cannabis will be sterilized prior to its distribution to eliminate any contamination with Aspergillus. Two varieties of cannabis will be utilized – mixed THC/CBD and placebo.

D.5.2 Facilities for the storage and security of cannabis: All cannabis will be locked in a double locked system in the SFGH Inpatient Pharmacy. They will also be stored in a double locked system in the CRC when released from the Inpatient Pharmacy to the ward for administration. The Inpatient Pharmacy is a locked facility to which only the pharmacy personnel have access. The cannabis will be stored in the designated locked freezer of the investigational drug refrigerator. In addition, the door of the locked freezer is connected to an audible alarm. The CRC has a locked medication room with a locked refrigerator and a locked drug cabinet. The cannabis will be stored in the freezer of the locked refrigerator. The CRC nursing staff will grind the cannabis for use in the Volcano vaporizer and load the vaporizer with the ground cannabis before patient use.

D.6 Statistical Considerations

This is a relatively small proof of principle evaluation designed to test feasibility and estimate effects of cannabanoid-based intervention to affect pain in patients with chronic persistent pain caused by SCD. In the primary project, we plan to study approximately 35 opioid-dependent patients (see Sample Size Considerations below). The design is the following:

Stage 1: Each patient will have an assessment of a baseline level of pain over a 5-day period with completion of a daily pain diary and, at the end of the 5 days, administration of comprehensive in-clinic interview using standardized questionnaires to assess their usual levels of pain.

Stage 2: Each patient who consents to continue participation in the study will be assigned three times a day use of inhaled vaporized cannabis for a period of 5 days. This will require a 5-day stay in the SFGH CRC The patient will continue use of the analgesics he/she customarily uses with additional analgesics on an as-needed basis during this 5-day period. The patient will again be asked to complete a daily pain diary. At the end of the 5 days, there will again be a comprehensive interview using standardized questionnaires to assess their levels of pain at the end of their clinic stay. The level of usage of opioids across the entire five days will be recorded, as well as the level of use on the last day.

D. 6.1 Data Analysis Plan: For each of the comprehensive assessments at the end of Stage 1 and Stage 2, a composite pain score, on a 0 to 100 scale, will be computed. The **primary outcome** of the study will be defined as the change in the composite pain score between the Stage 1 run-in period and the cannabinoid treatment period (Stage 2), that is,

Delta Pain = (Score after 5 days cannabinoid use) – (Score after 5 days of no use).

This design has the advantage that each patient acts as his/her own control, reducing the major within-person variability of the measurement. This design will also permit accurate determination of the level of opioid use during the 5-day stay.

D.6.2 Sample Size Considerations: We feel that a clinically meaningful change in the pain score would be a difference of 0.5 within-person standard deviations in the composite score (42). To detect a difference of this size at a significance level of 0.05 and statistical power of 80%, approximately 32 patients will need to be assessed. Assuming that 5% or fewer patients drop out before completing all Stages of the study, our target sample size for enrollment will be **35** patients with persistent chronic pain associated with SCD (and meeting other eligibility requirements).

D.6. 3 Statistical Analysis: The primary analysis associated with the self-reported pain-score outcome will be a paired t-test. Additional analyses taking into account baseline demographic factors (including gender and age) will be carried out using analysis of covariance. Similar considerations apply to analyses involving the levels of opioid use integrated across the 5-day stays and also levels on Day 5.

E. HUMAN SUBJECTS

E.1. Risk to Subjects

E.1.1 **Human subjects involvement and characteristics**: The study will enroll adult patients with sickle cell anemia and chronic pain on opioid analgesics. Subjects will be recruited from the San Francisco General Hospital Sickle Cell Clinic and from throughout the greater Northern California area. In the past, our cannabis investigations have attracted participants from across the United States.

E.1.2 Potential risks

1. The main side effects of inhaling cannabis with the Volcano vaporizer device would be cough and rarely bronchospasm. Other effects of cannabis inhalation include a feeling of being "high", anxiety, drowsiness, depression disorientation, paranoia, confusion, rapid heartbeat, palpitations, dizziness, fainting, redness of the eyeballs and dry mouth. The side effects may differ between the high THC and the high CBD strains as CBD is felt to be less psychoactive. Frequent cannabis smoking may be associated with an increased risk of chronic bronchitis, although other pulmonary problems are not increased. These are likely less with inhalation of vapors. Although few cannabis smokers develop dependence (<10%), some do. A cannabis withdrawal syndrome has been identified, but it is mild and short-lived. People experiencing withdrawal may exhibit some of the following symptoms: restlessness, irritability, mild agitation, difficulty sleeping, nausea and cramping.
2. Inhaling cannabis may affect drug metabolism, often in unpredictable ways. Therefore, a subject may experience less benefit from medications s/he is taking, or an increase in side effects associated with smoking. There are no scientific studies suggesting that cannabis decreases the benefits or increases

the side effects of other medications s/he may be taking. Prior studies have suggested an increased pain-relieving effect of opioids with vaporized cannabis.

3. Having blood drawn may result in bruises, which can be painful but carry no significant risks. The total amount of blood drawn will be about 7 tablespoons.
4. Inhaling cannabis may later be shown to be less effective in reducing pain or have more risks or side effects than is currently known. However, if the subject's pain is not well managed or if they have increased pain they will be allowed to manage their pain with whatever **supplemental analgesia** they normally use at home, except cannabis, and will be asked to bring their own supply to the CRC which will be dispensed by the CRC nurses as needed. In addition, the CRC nurses will evaluate subjects for oversedation. **The CRC will not prescribe additional medications. In the event of a pain crisis, the patient may be transferred to acute inpatient status**.
5. Remaining in the hospital for five days will interfere with usual routines and may become tedious.
6. Participation in research may cause a loss of privacy. In this study subjects will be asked about drug use and other possibly illegal activities. The researchers will keep information about each subject as confidential as possible, but complete confidentiality cannot be guaranteed. Subjects will be identified by a code. Subjects will not be personally identified in any publication about this study. However, records may be reviewed, under guidelines of the Federal Privacy Act, by the U.S. Food and Drug Administration (FDA); National Institute of Health (NIH), National Heart Lung and Blood Institute, and the research personnel from the Hematology-Oncology Division and the CRC at SFGH. The UCSF Committee on Human Research and other University of California personnel also may review or receive information about the subjects.
7. A Confidentiality Certificate will be requested from the Department of Health and Human Services (DHHS). This certificate will protect the study investigators from being forced to release any research data in which subjects are identified, even under a court order or subpoena. However, subjects may consent in writing to disclose identifying information, if they so choose. Subjects can request a copy of this certificate for their records.
8. The drug in this study may be unsafe for unborn babies. If female subjects are having sex that could lead to pregnancy, they must agree not to become pregnant. Women who are breast-feeding their baby may not join the study.
9. Subjects will have a positive urine test for cannabis following the study for approximately two weeks and even longer.
10. The risks of cannabidiol have not yet been fully documented. Though hardly conclusive, prior animal studies have suggested that clastogenicity and impaired spermatogenesis may result from CBD-rich cannabinoid use. We are thus requiring study participants to have prior experience with cannabidiol-rich cannabis.

E.2 Adequacy of Protection Against Risks

E.2.1 Recruitment and informed consent: Subjects will be recruited from the Hematology Service at SFGH, from other Bay Area and Northern California Sickle Cell Programs, and through notices or flyers on the UCSF campus and paid advertisements placed in the local print media. The study coordinator will arrange a screening visit with potential subjects.

E.2.2 Consent process and documentation: At the screening visit, prospective subjects will review the consent form in detail with the study coordinator who will answer all questions before inviting the patient to sign the consent form. It will be stated that participation in research is voluntary, and that subjects have the right to decline to participate or withdraw at any point in the study without jeopardy to their medical care. Subjects will also be asked to sign the UCSF Subject's Authorization for Research Access to Health Information and will be instructed that they may withdraw their authorization for this study to use their personal health information by contacting Dr. Abrams in writing to inform him of their decision. If subjects withdraw their authorization, the information already collected may continue to be used, to maintain the integrity of the study. If they choose not to sign this consent form, the investigator cannot use information from their medical records and they cannot participate in this research study. If a subject agrees to participate, the subject will sign the main consent form as well as the UCSF Subject's Authorization for Research Access to Health Information and a photocopy of the

signed consent forms with the Experimental Subjects' Bill of Rights will be given to the subject. The protocol will receive approval from the Institutional Committee on Human Research of the University of California, San Francisco prior to implementation.

E.2.3 Protection against risks: Participation in research may involve loss of privacy. The subjects' records will be handled as confidentially as is possible within the law. All records will be coded and stored in locked files. Copies of the signed consent forms are kept by the CRC, the subjects, and the principal investigator. No individual identities will be used in any reports or publications resulting from this investigation. However, records may be reviewed, under guidelines of the Federal Privacy Act, by the U.S. Food and Drug Administration (FDA); the National Institute of Health; National Heart Lung and Blood Institute and research personnel from the Hematology-Oncology Division at SFGH. A Confidentiality Certificate will be requested from the Department of Health and Human Services (DHHS). This certificate will protect the study investigators from being forced to release any research data in which a subject is identified, even under a court order or subpoena. However, a subject may consent in writing to disclose identifying information, if s/he so chooses. The specific measures to minimize each risk are described in the relevant sections above. In addition, there will be continuous safety surveillance with emphasis on the potential side effects of each procedure, as detailed above. Participation in the study will be discontinued if the subject fails to adhere to the study requirements in a way that may cause harm to him or herself or seriously interferes with the validity of the study results; or if the investigator determines that further participation would be detrimental to the subject's health or wellbeing.

Because of the nature of this study, subjects will not be given access to all of the health information gathered about them until the entire study is over. When the study is over, they may request access to all of the information the study has about them. In the event of a medical emergency or adverse event, their record will be made available to the treating physician to provide the best medical care.

Dr. Abrams will retain the research records, including information from their medical records, indefinitely for research purposes. However, their personal health information cannot be used for additional research without additional approval from either the subject or the review committee.

E.2.4 Data and Safety Monitoring Plan

Our Data Safety Monitoring Plan is designed to insure the safety of participants, the validity of data, and the appropriate termination of studies for which significant benefits or risks have been uncovered or when it appears that the trial cannot be concluded successfully. The progress of the trial and the safety of participants will be monitored regularly and frequently by the principal investigator. This will include weekly or biweekly assessments of data quality and timeliness, participant recruitment, accrual and retention, participant risk versus benefit, and other factors that can affect study outcome. Monitoring will also consider factors external to the study when interpreting the data, such as scientific or therapeutic developments that may have an impact on the safety of the participants or the ethics of the study.

Assessment of Risk: This study is considered to involve a low to moderate degree of risk to trial subjects. The cannabis cigarettes required for this study will be provided by the National Institute of Drug Abuse (NIDA) and prepared by the Research Triangle Institute of North Carolina. The cannabis will be sterilized prior to its distribution to eliminate any contamination with Aspergillus. The safety profile of inhaled vaporized cannabis is such that it is appropriate for the safety monitoring to be assumed by the investigator.

Anticipated Adverse Events: The side effects of inhaling vaporized cannabis include: cough, and rarely, bronchospasm. Acute side effects of smoked cannabis frequently include: tachycardia, redness of the eyeballs, depersonalization and dry mouth. Occasionally, side effects include palpitations, anxiety, euphoria and paranoia (43). Frequent cannabis smoking may be associated with an increased risk of lung illnesses, such as chronic bronchitis and changes in the cells of lungs, however inhalation of vaporized cannabis is associated with less risk. Smoking cannabis can significantly affect drug metabolism, often in unpredictable ways. Therefore, subjects may experience less benefit from medications that they are taking, or they may experience an increase in side effects associated with smoking. In our prior studies, however, we appreciated no significant alteration of concurrent medication blood levels and saw an increased analgesic effect of opioids

when used with vaporized cannabis. Severe or unexpected adverse drug reactions will be promptly reported to the IRB.

Safety Monitoring Plan: After completing a screening visit, subjects will be admitted to the CRC for five days. On Day 1 subjects will have blood drawn from markers of inflammation and sickle cell disease activity. Their one week pain diary will be collected. They will then be instructed to inhale vaporized cannabis using the uniform puff procedure, described by Foltin, in which the cannabis is inhaled once a minute until the desired dose is consumed. CRC nursing staff will observe all subjects while they vaporize. Subjects will be housed in a room with a fan ventilating to the outside. There will be continuous safety surveillance. The CRC nursing staff will monitor and assess the subject using the Drug Effects form and the Community Consortium Side Effects form and will monitor heart rate, blood pressure and respirations throughout the day. Participation in the study will be discontinued if the subject fails to adhere to the study requirements in a way that may cause harm to him or herself or may seriously interfere with the validity of the study results, or if the investigator determines that further participation would be detrimental to the subject's health or wellbeing.

Adverse Event Grading Scale: Adverse events will be graded based on the following general scale used by the Division of AIDS at the National Institute of Allergy and Infectious Diseases:

**Grade 1**	**Mild**	Transient or mild discomfort; no limitation in activity; no medical intervention/therapy required.
**Grade 2**	**Moderate**	Mild to moderate limitation in activity – some assistance may be needed; no medical intervention/therapy required.
**Grade 3**	**Severe**	Marked limitation in activity, some assistance usually required; medical intervention/therapy required, hospitalizations possible.
**Grade 4**	**Life- threatening**	Extreme limitation in activity, significant assistance required; significant medical intervention/therapy required, hospitalization or hospice care probable.

Reporting of Adverse Events: For the purpose of study monitoring and analysis, all Adverse Events (AE's) at a toxicity Grade 3 or higher associated with use of the study drug will be considered Serious Adverse Events (SAE's). All Serious Adverse Events will be recorded on the Division of AIDS Regulatory Operations Center Serious Adverse Experience form and reported to the U.S. Food and Drug Administration (FDA). Serious adverse experiences are defined as a subset of those adverse events (including deaths) that are possibly related to the study treatment and require reporting to the FDA. Internal reporting procedures have been developed for timely and accurate reporting of serious experiences in order to monitor subject safety, to comply with FDA regulations, and to disseminate safety information to our institutional review board. If a subject develops a Grade 4 serious adverse event they will be removed from the study treatment at that time. Subjects may withdraw from the trial at any time they wish.

Frequency of Safety Reviews: Since subjects will be hospitalized in the CRC at SFGH, the safety review will be done by the inpatient CRC nursing staff at each nursing shift and as part of an ongoing nursing assessment.

E.3 Potential benefits from proposed research to the subjects and others. The treatment study participants receive may prove to be more effective than other available treatment, but this cannot be guaranteed. If this is the case, then the study participants and individuals who have sickle cell anemia will benefit from this research. The main goal of the research, though, is to determine if inhaled cannabis is safe and effective when used as adjunctive therapy in combination with opioids to treat chronic pain in sickle cell patients and if it has any effect on inflammation and markers of disease progression.

E.4 Women and minority inclusion: See Targeted Enrollment Table.

E.4.1 Inclusion of women and minorities: There will be no exclusions based on gender or race.

E.4.2 Inclusion of children: Children will not be recruited for this study.

F. VERTEBRATE ANIMALS: Not Applicable

G. LITERATURE CITED

1. Claster S, Vichinsky EP. Managing sickle cell disease. BMJ. 2003 Nov 15;327(7424):1151-5.
2. Cichewicz DL. Synergistic interactions between cannabinoid and opioid analgesics. Life Sci. 2004;74(11):1317-24.
3. Smith FL, Cichewicz D, Martin ZL, Welch SP. The enhancement of morphine antinociception in mice by delta9-tetrahydrocannabinol. Pharmacol Biochem Behav. 1998;60(2):559-66.
4. Cichewicz DL, Martin ZL, Smith FL, Welch SP. Enhancement mu opioid antinociception by oral delta9-tetrahydrocannabinol: dose-response analysis and receptor identification. J Pharmacol Exp Ther. 1999;289(2):859-67.
5. Cichewicz DL, McCarthy EA. Antinociceptive synergy between delta(9)-tetrahydrocannabinol and opioids after oral administration. J Pharmacol Exp Ther. 2003;304(3):1010-5.
6. Committee on Advancing Pain Research, Care and Education Board on Health Sciences Policy. Relieving Pain in America: A Blueprint for Transforming Prevention, Care, Education, and Research. Institute of Medicine (IOM). 2011.
7. Taylor LEV, Stotts NA, Humphreys J, Treadwell MJ, Miaskowski C. A review of the literature on the multiple dimensions of chronic pain in adults with sickle cell disease. J Pain Symptom Manage. 2010;40:416-435.
8. Pugh G, Jr., Smith PB, Dombrowski DS, Welch SP. The role of endogenous opioids in enhancing the antinociception produced by the combination of delta 9-tetrahydrocannabinol and morphine in the spinal cord. J Pharmacol Exp Ther. 1996;279(2):608-16.
9. Welch SP, Stevens DL. Antinociceptive activity of intrathecally administered cannabinoids alone, and in combination with morphine, in mice. J Pharmacol Exp Ther. 1992;262(1):10-8.
10. Welch SP, Eads M. Synergistic interactions of endogenous opioids and cannabinoid systems. Brain Res. 1999;848(1-2):183-90.
11. Welch SP, Thomas C, Patrick GS. Modulation of cannabinoid-induced antinociception after intracerebroventricular versus intrathecal administration to mice: possible mechanisms for interaction with morphine. J Pharmacol Exp Ther. 1995;272(1):310-21.
12. Pugh G, Jr., Welch SP, Bass PP. Modulation of free intracellular calcium and cAMP by morphine and cannabinoids, alone and in combination in mouse brain and spinal cord synaptosomes. Pharmacol Biochem Behav. 1994;49(4):1093-100.
13. Kaymakcalan S. Pharmacological similarities and interactions between cannabis and opioids. Adv Biosci. 1978;22-23:591-604.
14. Physicians' desk reference : PDR. Oradell, N.J.: Medical Economics Co.:v.
15. Warfield CA. Controlled-release morphine tablets in patients with chronic cancer pain: a narrative review of controlled clinical trials. Cancer. 1998;82(12):2299-306.
16. Davis MP, Varga J, Dickerson D, Walsh D, LeGrand SB, Lagman R. Normal-release and controlled-release oxycodone: pharmacokinetics, pharmacodynamics, and controversy. Support Care Cancer. 2003;11(2):84-92.
17. Leow KP, Cramond T, Smith MT. Pharmacokinetics and pharmacodynamics of oxycodone when given intravenously and rectally to adult patients with cancer pain. Anesth Analg. 1995;80(2):296-302.
18. Curtis GB, Johnson GH, Clark P, et al. Relative potency of controlled-release oxycodone and controlled-release morphine in a postoperative pain model. Eur J Clin Pharmacol. 1999;55(6):425-9.

19. Heiskanen T, Kalso E. Controlled-release oxycodone and morphine in cancer related pain. Pain. 1997;73(1):37-45.

20. Bruera E, Belzile M, Pituskin E, et al. Randomized, double-blind, cross-over trial comparing safety and efficacy of oral controlled-release oxycodone with controlled-release morphine in patients with cancer pain. J Clin Oncol. 1998;16(10):3222-9.

21. Maddocks I, Somogyi A, Abbott F, Hayball P, Parker D. Attenuation of morphine-induced delirium in palliative care by substitution with infusion of oxycodone. J Pain Symptom Manage. 1996;12(3):182-9.

22. Hagen NA, Babul N. Comparative clinical efficacy and safety of a novel controlled-release oxycodone formulation and controlled-release hydromorphone in the treatment of cancer pain. Cancer. 1997;79(7):1428-37.

23. Hall W. The health risks of cannabis. Aust Fam Physician. 1995;24(7):1237-40.

24. Kalant H. Adverse effects of cannabis on health: an update of the literature since 1996. Prog Neuropsychopharmacol Biol Psychiatry. 2004;28(5):849-63.

25. Hart CL, van Gorp W, Haney M, Foltin RW, Fischman MW. Effects of acute smoked marijuana on complex cognitive performance. Neuropsychopharmacology. 2001;25(5):757-65.

26. Borg J, Gershon S, Alpert M. Dose effects of smoked marihuana on human cognitive and motor functions. Psychopharmacologia. 1975;42(3):211-8.

27. Wilson WH, Ellinwood EH, Mathew RJ, Johnson K. Effects of marijuana on performance of a computerized cognitive-neuromotor test battery. Psychiatry Res. 1994;51(2):115-25.

28. Heishman SJ, Arasteh K, Stitzer ML. Comparative effects of alcohol and marijuana on mood, memory, and performance. Pharmacol Biochem Behav. 1997;58(1):93-101.

29. Chait LD, Perry JL. Factors influencing self-administration of, and subjective response to, placebo marijuana. Behav Pharmacol. 1992;3(6):545-552.

30. Johnstone RE, Lief PL, Kulp RA, Smith TC. Combination of delta9-tetrahydrocannabinol with oxymorphone or pentobarbital: Effects on ventilatory control and cardiovascular dynamics. Anesthesiology. 1975;42(6):674-84.

31. Naef M, Curatolo M, Petersen-Felix S, Arendt-Nielsen L, Zbinden A, Brenneisen R. The analgesic effect of oral delta-9-tetrahydrocannabinol (THC), morphine, and a THC-morphine combination in healthy subjects under experimental pain conditions. Pain. 2003;105(1-2):79-88.

32. A. Klein, T. Cannabinoid-based drugs as anti-inflammatory therapeutics. Nature Reviews Immunology 5, 400-411, May 2005.

33. Izzo AA, Borrelli F, Capasso R, DiMarzo V, Mechoulam R. Non-psychotropic plant cannabinoids: new therapeutic opportunities from an ancient herb. Trends in Pharmacological Sciences. 2009;30:515-527.

34. Booz GW. Cannabidiol as an emergent therapeutic strategy for lessening the impact of inflammation on oxidative stress. Free Radical Biology and Medicine. 2011;51:1054-1061.

35. Abrams DI, Hilton JF, Leiser RJ, et al. Short-term effects of cannabinoids in patients with HIV-1 infection: a randomized, placebo-controlled clinical trial. Ann Intern Med. 2003;139(4):258-66.

36. Bredt BM, Higuera-Alhino D, Shade SB, Hebert SJ, McCune JM, Abrams DI. Short-term effects of cannabinoids on immune phenotype and function in HIV-1-infected patients. J Clin Pharmacol. 2002;42(11 Suppl):82S-89S.

37. Kosel BW, Aweeka FT, Benowitz NL, et al. The effects of cannabinoids on the pharmacokinetics of indinavir and nelfinavir. AIDS. 2002;16(4):543-50.

38. Abrams DI, Vizoso HP, Shade SB, Jay C, Kelly ME, Benowitz N. Vaporization as a smokeless cannabis delivery system: A pilot study. Clinical Pharmacology and Therapeutics, 82:572-578, 2007.

39. Abrams DI, Jay C, Shade S, Vizoso H, Reda H, Press S, Kelly ME, Rowbotham M, Petersen K. Cannabis in painful HIV-associated sensory neuropathy: a randomized, placebo-controlled trial. Neurology 68:515-521, 2007.

40. Abrams DI, Couey P, Shade SB, Kelly ME, Benowitz NL. Cannabinoid:opioid interaction in chronic pain. Clinical Pharmacology and Therapeutics 90:844-851, 2011.

41. Foltin RW, Fischman MW, Byrne MF. Effects of smoked marijuana on food intake and body weight of humans living in a residential laboratory. Appetite. 1988;11(1):1-14.

42. Dupont WD, Plummer WD, Jr. Power and sample size calculations. A review and computer program. Control Clin Trials. 1990;11(2):116-28.

43. Taylor HG. Analysis of the medical use of marijuana and its societal implications. J Am Pharm Assoc (Wash). 1998;38(2):220-7.
