# Supplementary material for: Effect of Inhaled Cannabis for Pain in Adults With Sickle Cell Disease: A Randomized Clinical Trial
Source: JAMA Netw Open. 2020 Jul 17;3(7):e2010874. doi: 10.1001/jamanetworkopen.2020.10874 (PMC7368173; doi:10.1001/jamanetworkopen.2020.10874)
Supplement: Supplement 2. — eFigure. Forest Plot [file jamanetwopen-3-e2010874-s002.pdf]

Supplementary Online Content

Abrams DI, Couey P, Dixit N, et al. Effect of inhaled cannabis for pain in adults with sickle cell disease: a randomized clinical trial. *JAMA Netw Open*. 2020;3(7):e2010874. doi:10.1001/jamanetworkopen.2020.10874

eFigure. Forest Plot

This supplementary material has been provided by the authors to give readers additional information about their work.

© 2020 Abrams DI et al. *JAMA Network Open.*

eFigure. Forest Plot

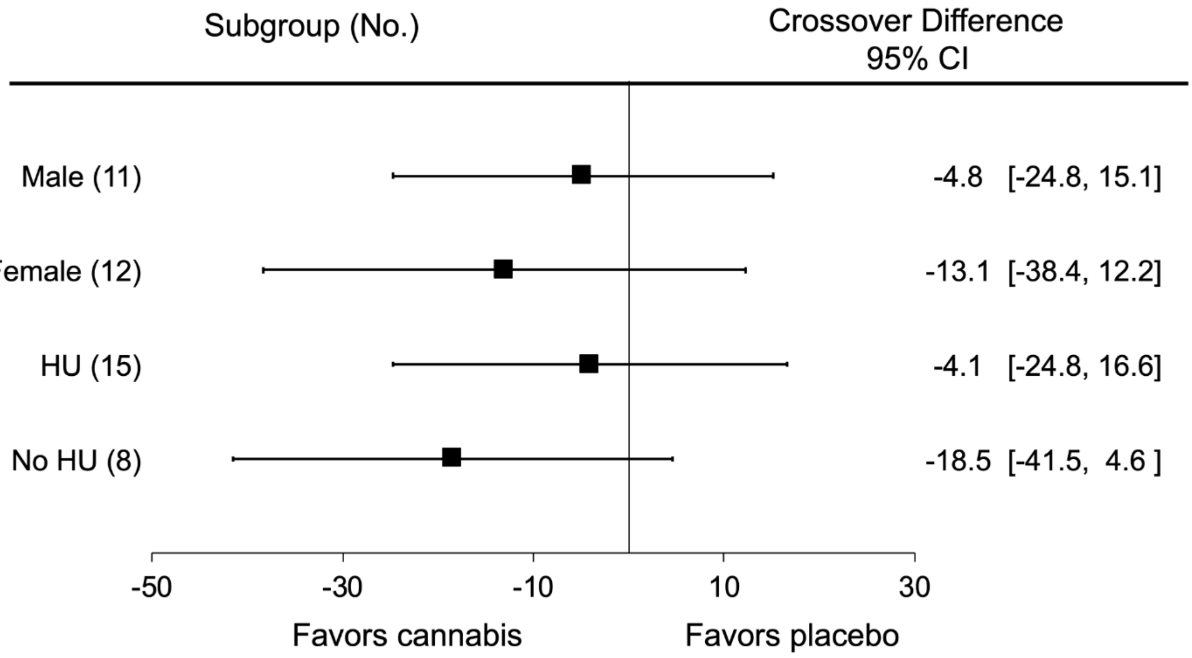

The within patient difference in pain rating between cannabis and placebo treatment, referred to as the crossover difference, was averaged over all five days of treatment. The average crossover difference and 95% confidence interval are shown by gender and hydroxyurea use. A negative crossover difference favors cannabis treatment, while a positive crossover difference favors placebo treatment.

© 2020 Abrams DI et al. *JAMA Network Open.*
