# Supplementary material for: Effect of Inhaled Cannabis for Pain in Adults With Sickle Cell Disease: A Randomized Clinical Trial
Source: JAMA Netw Open. 2020 Jul 17;3(7):e2010874. doi: 10.1001/jamanetworkopen.2020.10874 (PMC7368173; doi:10.1001/jamanetworkopen.2020.10874)
Supplement: Supplement 3. — Data Sharing Statement [file jamanetwopen-3-e2010874-s003.pdf]

Data Sharing Statement

Abrams. Effect of Inhaled Cannabis for Pain in Adults With Sickle Cell Disease. *JAMA Netw Open*. Published July 17, 2020. 10.1001/jamanetworkopen.2020.10874

Data
Data available: Yes
Data types: Deidentified participant data
How to access data: John Connett john-c@ccbr.umn.edu
When available: With publication

Supporting Documents
Document types: Informed consent form
How to access documents: Paul.Couey@ucsf.edu
When available: With publication

Additional Information
Who can access the data: Researchers whose proposed use of data has been approved
Types of analyses: For approved purposes
Mechanisms of data availability: After approval of a proposal
Any additional restrictions: None
